# The Genetic Architecture of the Genome-Wide Transcriptional Response to ER Stress in the Mouse

**DOI:** 10.1371/journal.pgen.1004924

**Published:** 2015-02-04

**Authors:** Clement Y Chow, Xu Wang, David Riccardi, Mariana F. Wolfner, Andrew G. Clark

**Affiliations:** Department of Molecular Biology and Genetics, Cornell University, Ithaca, New York, United States of America; Dartmouth College, UNITED STATES

## Abstract

Endoplasmic reticulum (ER) stress occurs when misfolded proteins accumulate in the ER. The cellular response to ER stress involves complex transcriptional and translational changes, important to the survival of the cell. ER stress is a primary cause and a modifier of many human diseases. A first step to understanding how the ER stress response impacts human disease is to determine how the transcriptional response to ER stress varies among individuals. The genetic diversity of the eight mouse Collaborative Cross (CC) founder strains allowed us to determine how genetic variation impacts the ER stress transcriptional response. We used tunicamycin, a drug commonly used to induce ER stress, to elicit an ER stress response in mouse embryonic fibroblasts (MEFs) derived from the CC founder strains and measured their transcriptional responses. We identified hundreds of genes that differed in response to ER stress across these genetically diverse strains. Strikingly, inflammatory response genes differed most between strains; major canonical ER stress response genes showed relatively invariant responses across strains. To uncover the genetic architecture underlying these strain differences in ER stress response, we measured the transcriptional response to ER stress in MEFs derived from a subset of F1 crosses between the CC founder strains. We found a unique layer of regulatory variation that is only detectable under ER stress conditions. Over 80% of the regulatory variation under ER stress derives from *cis*-regulatory differences. This is the first study to characterize the genetic variation in ER stress transcriptional response in the laboratory mouse. Our findings indicate that the ER stress transcriptional response is highly variable among strains and arises from genetic variation in individual downstream response genes, rather than major signaling transcription factors. These results have important implications for understanding how genetic variation impacts the ER stress response, an important component of many human diseases.

## Author Summary

Genetic variation among individuals can greatly impact the severity of disease outcomes. To understand the effects of different genetic backgrounds on disease presentation, we focused on ER stress, an important cellular stressor that impacts many human diseases. We examined how genetic variation affects ER stress response, at the RNA level, in eight laboratory mouse strains and their hybrid progeny. We find that each mouse strain responds in a unique way to ER stress, and we characterized the patterns of genetic variation that underlie the differences in ER stress response between the strains. We find that the strains show major differences in their inflammatory response to ER stress, a critical component to disease. The results of this study are important for understanding potential ways in which genetic variation in ER stress response could impact disease, and lays the groundwork for future studies in human patients.

## Introduction

The endoplasmic reticulum (ER) is a large cellular organelle that is involved in protein processing, lipid metabolism, and calcium storage. ER stress occurs when misfolded proteins accumulate in the lumen of the ER [1]. A cell responds to ER stress with the unfolded protein response (UPR), which consists of three main signaling branches, IRE1, ATF6 and PERK [1]. The UPR returns the ER to homeostasis by inducing the expression of chaperones and other proteins involved in refolding or degrading misfolded proteins. If ER stress cannot be resolved, the cell will initiate a program of apoptosis, leading to cell death [1].

ER stress is a critical component of many diseases. In some cases, altered ER stress responses can be a primary cause of disease [2]. However, more commonly the response to ER stress is an important modifier of disease severity [2]. Numerous studies have demonstrated that altering ER stress responses in the mouse, either pharmacologically or genetically, has profound effects on disease outcome. This has been demonstrated in different diseases, such as type 2 diabetes, certain forms of cancer, and neurodegenerative diseases. For example, in various mouse models of diabetes, genetic manipulation of *CHOP*, a pro-apoptotic factor mediated by the PERK pathway, prevents ER stress-induced β cell death, resulting in more normal glucose homeostasis [3, 4]. In a form of familial amyotrophic lateral sclerosis, a neurodegenerative motor neuron disease caused by mutations in *SOD1*, ER stress pathways are activated, but different genetic alterations to the PERK pathway can ameliorate [5] or accelerate [6] the disease. These experimental genetic examples are a proof-of-principle that genetic variation in ER stress response may modify the outcome of certain diseases.

While the ER stress response pathway is well studied, it is only recently that we are beginning to understand how genetic variation impacts an individual’s response to ER stress. ER stress-responsive gene expression is variable among immortalized human B cells and this variation is likely heritable [7, 8]. We have recently shown that ER stress responsive gene expression is also highly variable across wild-derived, inbred *Drosophila melanogaster* strains, and that susceptibility to ER stress in these strains is associated with SNPs in canonical ER stress genes, such as *Xbp1*, as well as many novel candidate ER stress genes [9]. Although one need not be surprised that there is inter-individual variability in ER stress response, these studies lay the foundation for understanding how genetic variation in novel and known components of ER stress response modulate the overall response of the cell to an overload of misfolded proteins.

Identifying the variable elements of the ER stress response is a critical first step in understanding how ER stress responses impact disease. Studying variation in ER stress response also provides the opportunity to nominate and eventually functionally validate new genes that influence the ER stress response, genes that may be missed by studying only one inbred laboratory strain [9]. Studies of human ER stress variation are limited to immortalized cell lines [7, 8] and cannot be extended to *in vivo* studies. The mouse, however, is uniquely suited for understanding the genetic variation in ER stress response, both in cultured cell lines as well as *in vivo*, and allows for direct extension to and testing in models of human disease.

Tools for studying genome-wide genetic variation in the mouse are now becoming widely available. For example, the mouse Collaborative Cross (CC) is a large panel of new recombinant-inbred (RI) strains that are derived from eight existing laboratory and wild-derived strains [10]. Together these eight founder strains capture ~90% of the known genetic variation, including SNPs and structural variants, in all available mouse strains [10] and the amount of genetic variation present mirrors that of the human population [11, 12, 13]. Through a carefully designed breeding scheme, each RI strain equally captures a randomized portion of the genomes of each of the eight founder strains [14]. After scoring genotypes of these RI strains, rapid progress in the study of systems genetics and complex traits can be made by phenotypic analysis [14, 15].

To study the extent and genetic architecture of ER stress transcriptional response variation in the mouse, we took advantage of the genetic diversity in the eight founder strains employed in the CC. To identify the genes that are most variable in their transcript-level response to ER stress, we used tunicamycin to induce ER stress in mouse embryonic fibroblasts (MEFs) derived from the CC founder strains. We found that hundreds of genes vary among strains in their ER stress-induced transcript-level responses. Strikingly, the most variable response genes are enriched in inflammation-related transcription factor binding sites and in functions related to inflammatory response and are not major, recognized canonical ER stress genes. To uncover the genetic architecture underlying these strain differences in ER stress-induced gene expression, we measured gene expression in ER stress treated MEFs from a subset of F1 crosses of the eight CC founder strains. We show that variation in ER stress response is controlled by a unique genetic architecture that is not detectable under healthy conditions. The bulk of strain differences in ER stress-induced gene expression derive from differences in *cis*-regulatory control, rather than differences in *trans*-regulatory control. We also find significant effects of ER stress on changes in allele-specific responses on key ER stress response genes. Together these data suggest that there is a decipherable genetic network controlling differences in a basic cellular response like ER stress. Our results also may have important implications for mouse genetic background selection, identifying disease modifiers, and understanding the plasticity of the ER stress response.

## Results and Discussion

### ER stress-induced expression across genetically diverse mouse strains

**Figure 1:**
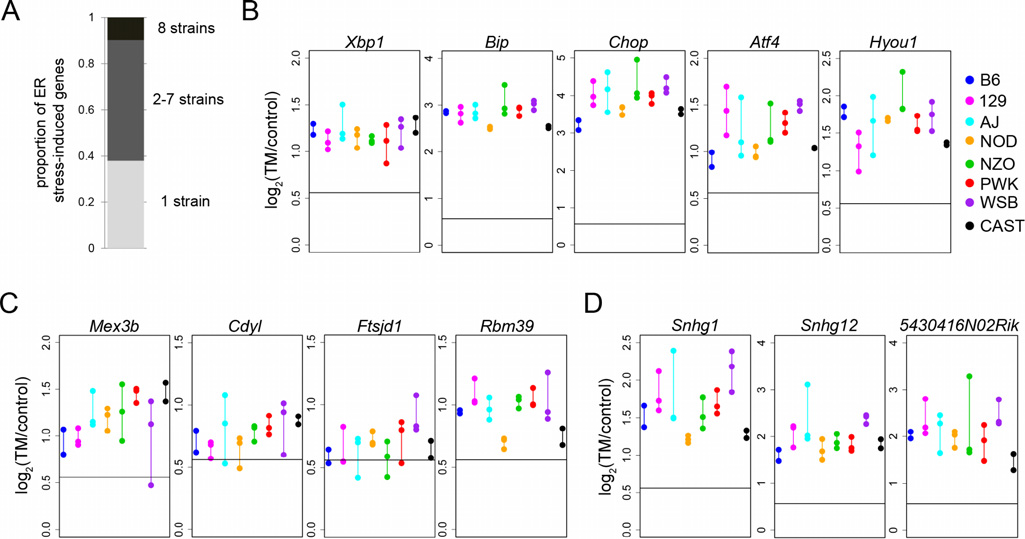
ER stress-induced genes. (A) Proportion of ER stress-induced genes that are shared among strains and genes that are uniquely upregulated in only one strain. Change in gene expression is displayed for common ER stress-induced genes with known ER stress functions (B), genes with functions that regulate gene expression (C), and noncoding RNAs (D). For each strain (different colors), all biological replicates are displayed as points connected by a vertical line (B-D). Horizontal line represents 1.5 fold change (B-D). TM: tunicamycin.

To evaluate the extent of genetic variation in ER stress-induced gene expression, MEFs derived from the eight founder strains of the CC [10] were exposed to tunicamycin (TM) (or DMSO control). TM inhibits glycosylation, causing the accumulation of misfolded proteins in the lumen of the ER, triggering a robust ER stress response (from here forward, ER stress refers to TM-induced ER stress). TM is a commonly used pharmacological agent used to experimentally induce ER stress in MEFs [16] and produces a strong transcriptional response to ER stress that models the ER stress that can occur during disease. MEFs were exposed to 2ug/ml of TM for four hours. This concentration and exposure time is sufficient to induce a strong ER stress transcriptional response, while secondary effects of TM are still absent. Following TM exposure, cells were harvested and RNA was subjected to RNAseq. For each strain, ER stress-induced gene expression in TM-treated cells was compared to expression from DMSO-treated control cells. We defined an ER stress-induced gene as one whose RNA levels are upregulated after TM exposure by at least 1.5 fold (FDR 1%) in at least one of the eight strains. By this criterion, there were a total of 2,182 ER stress-induced genes in these eight mouse strains (S1 Table; Fig. 1A). Among the ER stress-induced genes, 214 (10%) are upregulated in all eight strains and are designated ‘common ER stress-induced genes’. The set of common ER stress-induced genes shows enrichment for ER stress function (e.g. GO:0006986: response to unfolded protein, *q* = 1.45×10^-4^ and GO:0034976: response to endoplasmic reticulum stress, *q* = 0.048; Table 1). Some of these common induced genes include canonical ER stress genes such as *Xbp1*, *CHOP* (*Ddit3*), *Bip* (*Hspa5*), *Atf4*, and *Hyou1* (Fig. 1B). Additionally, common ER stress-induced genes are enriched for genes with the NFYA and C/EBPα transcription factor binding sites (Table 2). NFYA interacts with the ER stress transcription factor ATF6, to bind to the ER stress responsive elements, ERSE and ERSEII [17, 18]. C/EBPα interacts with CHOP, a PERK/ATF4 induced transcription factor, under ER stress. Strikingly, we did not observe enrichment for functions related to apoptosis and cell death, indicating that apoptosis signaling has not been initiated in the MEFs. The enrichment of canonical ER stress genes in the set of common ER stress-induced genes is a ‘proof-of-principle’ that TM sufficiently induces the ER stress response in all eight strains and that observations made from these studies reflect strong ER stress responses.

**Table 1:** GO analysis.

	**GO**	**Term**	**fold enrichment**	***p***	***q***
**ER stress induced genes**	GO:0051789	response to protein stimulus	10.4	9.0×10^-8^	0.00011
	GO:0006986	response to unfolded protein	13.7	2.5×10^-7^	0.00014
	GO:0006350	Transcription	2.0	9.7×10^-6^	0.00381
	GO:0045449	regulation of transcription	1.7	0.00011	0.03194
	GO:0006984	ER-nuclear signaling pathway	16.5	0.00021	0.04872
	GO:0034976	response to endoplasmic reticulum stress	15.8	0.00025	0.04756
**ER stress induced genes with strain effect**	GO:0005125	cytokine activity	6.2	4.3×10^-9^	1.5×10^-6^
	GO:0008009	chemokine activity	14.8	1.2×10^-7^	2.1×10^-5^
	GO:0042379	chemokine receptor binding	14.4	1.5×10^-7^	1.7×10^-5^
	GO:0008083	growth factor activity	4.9	8.7×10^-5^	0.00756

**Table 2:** Transcription factor binding site analysis.

**ER stress induced genes**						
TF	Z-score	Fisher score	Target gene hits	Target gene non-hits	Background gene hits	Background gene non-hits
NFYA	22.75	1.94E-03	76	103	4824	10326
ReL	22.35	3.04E-03	111	68	7798	7352
NF-kB	16.76	3.24E-03	89	90	5960	9190
ELK1	11.7	1.44E-02	140	39	10697	4453
C/EBPα	14.44	1.67E-02	113	66	8322	6828
NF-kB1	16.78	4.59E-02	43	136	2832	12318
**ER stress induced genes with strain effect**						
TF	Z-score	Fisher score	Target gene hits	Target gene non-hits	Background gene hits	Background gene non-hits
ReLA	32.77	0.01115	37	46	4846	10304
NF-kB1	23.04	0.02924	23	60	2832	12318

Among the common ER stress-induced genes are some genes with no previously known function in the ER stress response. These putative ER stress genes fall into diverse categories. In some cases, genes not previously implicated in ER stress response have functions in processes that are important to the ER stress response. These include genes involved in Golgi trafficking (i.e. *Rab39b*), oxidoreductase activity (*Oxnad1*), and energy metabolism (*Cpox*). This co-occurrence of novel putative ER stress genes with canonical ER stress genes, across all eight strains, suggests that these response genes might be important in ER stress and warrant future study.

In addition to the enrichment of ER stress-related functions, common ER stress-induced genes are also enriched for functions involved in transcription (GO:0006350: transcription − *q* = 0.0038 and GO:0045449: regulation of transcription − *q* = 0.0319; Table 1). Forty-seven of the 214 common ER stress-induced genes are involved in the ‘regulation of transcription’ (GO:0045449). Some of these genes encode known ER stress transcription factors such as Xbp1 and Atf4. However, other transcription factors, like Arid5a, have no previously known role in ER stress. Even more striking, at least 15 of these transcription factors are unstudied zinc-finger proteins with no known function (e.g. Zfp191 and Zfp202). This enrichment for transcription factors with unknown function reinforces the idea that that the ER stress response involves a complex network of gene regulation, as condition-dependent elevation of transcription factor expression nearly always stimulates expression of downstream target genes as well [19].

A large component of the ER stress response involves changes in gene expression through different mechanisms including transcriptional regulation, RNA degradation, and sequestration of RNAs to stress granules [19]. In addition to the enrichment in transcription-related functions, we also identified genes that regulate gene expression through other mechanisms. Some of these genes function in chromatin remodeling (*Cdyl*), RNA binding (*Mex3b*), and RNA metabolism (*Ftsjd1* and *Rbm39*) (Fig. 1C). Non-coding RNAs (ncRNAs) are contributors to another mechanism of regulating gene expression [20]. We also identified a number of ncRNAs that are upregulated across all eight strains by ER stress. These include the lincRNAs *Snhg1* and *Snhg12* and other ncRNAs like *2410006H16Rik*, *5430416N02Rik*, and *9430008C03Rik* (Fig. 1D). Little is known of the exact role of ncRNAs in regulating the ER stress response. However, recent studies have suggested that ncRNAs might be important in fine-tuning the ER stress response. For example, the ncRNA *Gadd7* regulates reactive oxygen species induced ER stress through a feed-forward loop [21] and certain miRNAs are now known to be regulated by ER stress [22, 23, 24]. For the most part, however, the mechanisms of these actions are unknown. The fact that these ncRNAs are all upregulated over two-fold in response to ER stress across eight diverse mouse strains, suggests that they may play an important role in ER stress response.

### ER stress-induced expression is variable among mouse strains

The eight genetically diverse founder strains of the CC provide the opportunity to identify the genes that display variation in ER stress-induced expression. Due to the strong conservation of the ER stress response, studying these genes in the mouse may provide clues as to which portions of the response network can be subject to variation in the human population. In fact, most ER-stress induced genes are not upregulated in all eight strains. Of the 2,182 ER stress-induced genes, 829 (38%) are uniquely upregulated by ER stress in only one of the eight strains. 1139 genes (52%) showed shared up-regulation in two to seven strains (S1 Table; Fig. 1A).

**Figure 2:**
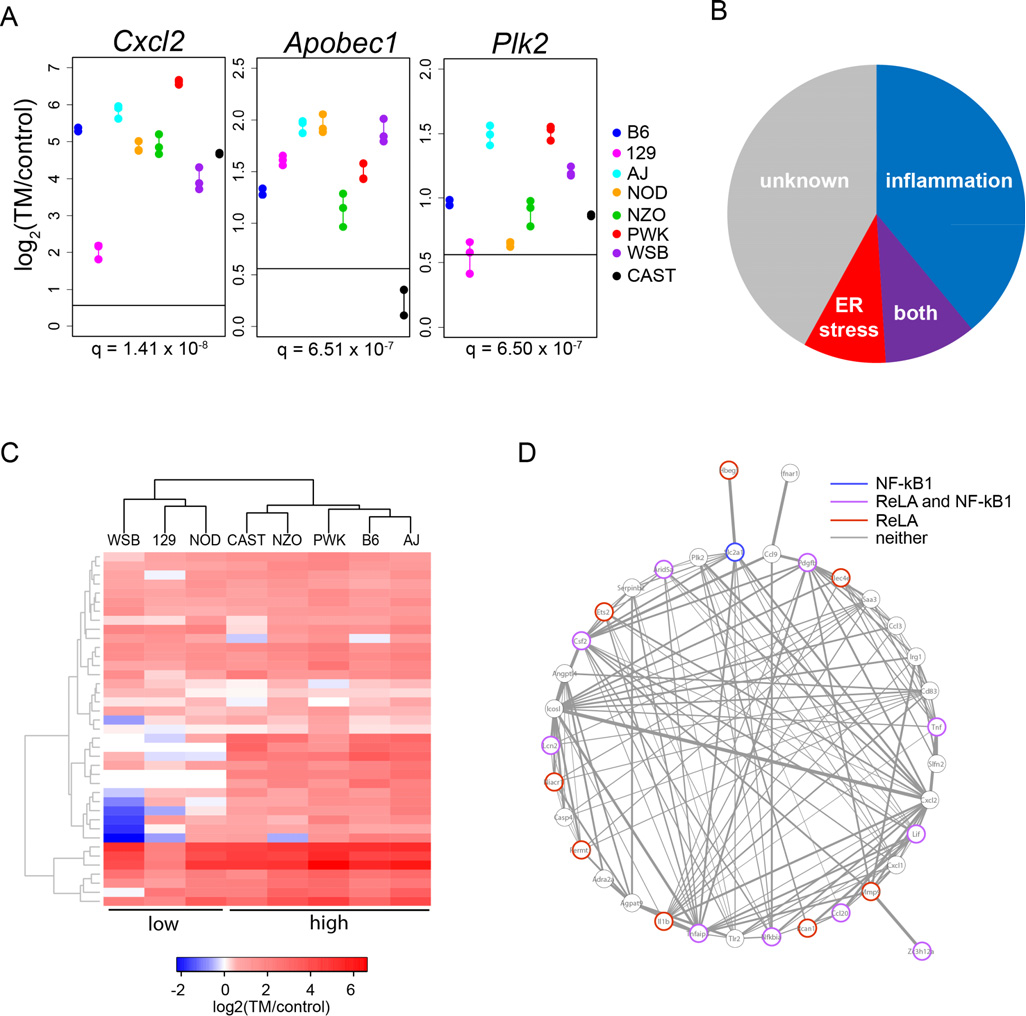
ER stress-induced genes with a strain effect are enriched for inflammation functions. (A) The top three genes with the strongest strain effect. Horizontal line represents 1.5 fold. For each strain (different colors), all biological replicates are displayed as points connected by a vertical line. Horizontal line represents 1.5 fold; *q* = FDR correction of the strain effect. (B) Literature search identifies the proportion of genes with inflammation functions, ER stress functions, inflammation and ER stress functions (both), or unknown ER stress functions in the 100 genes with the strongest strain effect (FDR <0.01%). (C) Cluster analysis indicates that strains are either in a low (WSB, 129, and NOD) or high (CAST, NZO, PWK, B6, and A) inflammation group. Cluster analysis was performed with the 39 inflammation genes among the 100 genes with the strongest strain effect. For gene identities see S1 Fig. (D) Correlation network showing that inflammation genes are highly correlated in their response to ER stress. Edge thickness represents the correlation coefficient between the two nodes. Only significant pairwise correlations are displayed (Spearman’s; *P* < 0.05). Genes that contain ReLA and NF-kB1 transcription factor binding sites are color coded. All pairwise correlations can be found in S4 Table. TM: tunicamycin.

To identify the genes with the most variable ER stress-induced expression among strains, gene expression results were tested using linear models to assess differences among the eight strains. 14.5% of ER stress-induced genes (317/2182 genes) show a significant strain effect on their induced expression (FDR 1%, S2 Table). Strikingly, the ten genes with the most significant strain effect all have very clear roles in inflammation (in order of statistical significance of strain effect: *Cxcl2*, *Apobec1*, *Plk2*, *Agpat9*, *Ccl20*, *Pdgfb*, *Lif*, *Cxcl1*, *Mmp9*, and *Clec4e*; Fig. 2A). GO analysis of the ER stress-induced genes with a strain effect further demonstrated a strong enrichment for inflammation functions. The three most significant GO categories are cytokine activity (GO:0005125: *q* = 1.49×10^-6^), chemokine activity (GO:0008009: *q* = 2.09×10^-5^), and chemokine receptor binding (GO:0042379: *q* = 1.73×10^-5^) (Table 1). We did not find enrichment of genes specifically involved in apoptosis signaling. Genes showing the most variable ER-stress-induced expression are also enriched for the inflammation related transcription factor binding sites, RelA and NF-kB1 (Table 2), but are not enriched for transcription factor binding sites related to ER stress signaling. The RelA and NF-kB1 transcription factors are members of the NF-κB family of transcription factors and dimerize in various combinations to control gene expression in response to stimuli such as inflammation [25]. In fact, we find that transcript levels of four members of the NF-κB family of transcription factors show significant variation among strains: *Nfkb1* (*q* = 0.009; encoding NF-kB1), *Nfkb2* (*q* = 0.003; encoding NF-kB2), *Rela* (*q* = 0.03; encoding RelA), and *Relb* (*q* = 0.002; encoding RelB) (S2 Table).

A literature search indicates that 39 of the top 100 genes with the most significant strain effect (FDR <0.01%), have very clear roles in inflammation, 9 have known roles in ER stress response (i.e. *Atf3* and *Derl3*), 10 have roles in both inflammation and ER stress, and 42 have no known function in either inflammation or ER stress (S3 Table; Fig. 2B). Cluster analysis with the 39 inflammation genes indicates that the eight strains fall into either a low (WSB, 129, and NOD) or high (CAST, NZO, PWK, B6, and A) inflammation response group (S1 Fig.; Fig. 2C). To understand how these inflammation genes might function within a network, we performed a correlation analysis. These 39 inflammation genes function in a highly correlated network. Half contain either a RelA or a NF-kB binding site, or both (S4 Table; Fig. 2D).

ER stress response and inflammation are interconnected in complex ways. ER stress can induce and can be a consequence of inflammatory responses. ER stress induces NF-kB signaling by various routes. Ire1 activation during ER stress results in the degradation of IkBa, an inhibitor of NF-kB signaling, resulting in increased NF-kB signaling [26]. PERK activation during ER stress results in global translation attenuation, which in turn results in decrease of IkBa production and increased NF-kB signaling [26]. Recent evidence suggests that Atf6 can also influence NF-kB signaling, but the exact role of Atf6 in this process is still unclear [27]. Because we do not find variation in the expression levels of key ER stress genes like *Ire1*, *PERK* or *Atf6*, it is likely that the variation we observe originates from genes encoding effectors of NF-kB signaling or NF-kB transcription factors themselves. The enrichment in NF-kB binding sites and the strong correlations among the inflammation genes further support this. More extensive work will be needed to identify which are the primary variable response genes and which are secondary. Nevertheless, inflammatory response to ER stress appears to be an important difference between strains and might be an attribute of stress response that is also variable in humans. The intersection between inflammation and ER stress might be a fruitful avenue for identifying important modifiers of human disease.

### Inference of *cis*-regulatory variation in ER stress response

The fact that there is variation in ER stress-induced transcript levels among eight genetically diverse mouse strains suggests that there is genetic variation underlying these differences. In order to understand how genetic variation impacts the ER stress response, we need to know how genetic variation influences gene expression differences among strains under non-stressed conditions and how it changes once ER stress is applied. Transcriptional variation may arise from polymorphisms in one (or a few) transcription factors (*trans*-), or each response gene could harbor polymorphisms affecting its own expression (*cis*-).

The use of F1 crosses in this study was specifically designed to allow us to quantify the magnitude of *cis*-regulatory variation and to partition the inter-strain variance in expression into its *cis*- and *trans*- components [28, 29, 30, 31]. For genes that show an expression difference between two strains, identification of *cis*- and *trans*-regulatory differences requires comparing the relative allelic expression in the F1 to the relative expression of the parental strains [28, 29, 30, 31]. In the F1, the two parental alleles are exposed to the same *trans*- factors in the same levels and combinations. Thus, the ratio of allelic expression is a direct measure of *cis*-regulatory differences between parental strains—if the allelic ratio matches the ratio of the parental expression levels, the expression difference is attributed to *cis*-regulation. If the allelic ratio differs from the ratio of parental expression levels, then the difference is attributable to *trans*-regulation [29]. We focused on five F1 crosses. In all five crosses, B6 was the maternal strain and the paternal strain in each cross was one of five CC founder strains (129, NOD, NZO, WSB, and CAST; see materials and methods for F1 cross abbreviations). B6 was maintained as the maternal strain in all F1 crosses to avoid parent-of–origin and imprinting effects, but future studies with reciprocal crosses are warranted, as nothing is known about how parent-of-origin and imprinting influence ER stress responses. MEFs from each F1 cross were exposed to control and ER stress conditions, and transcript levels were measured by RNA-seq.

*cis-/trans*- analysis can only be performed for a gene that has sufficiently high expression and that harbors SNPs within the transcribed region that discriminate between parental alleles. In this study, we only considered informative genes with at least two such SNPs. The number of informative genes for analysis ranged from 2246 to 7,954 (xNOD: 2246, x129: 2843, xNZO: 2910, xWSB: 3285, xCAST: 7954; S5 Table). For the rest of this analysis, unless otherwise noted, focus will mainly be on the xCAST F1 cross because this combination has the largest number of informative genes.

### ER stress does not change the general pattern of regulatory divergence

The regulatory control of ER stress-induced expression is complex. The three arms of the UPR all induce major transcription factors (e.g. Xbp1, Atf6, and Atf4) and those transcription factors can activate other transcription factors [1, 19]. A polymorphism that affects the function of one of these would result in many *trans*-regulatory differences in their targets genes. On the other hand, some target genes contain ER stress responsive elements (ERSREs) that act as binding sites for these transcription factors [18, 32, 33, 34, 35]. A polymorphism in an ERSRE or other regulatory element could result in a *cis*-regulatory difference among strains for a particular target gene. To identify which of these possibilities contributes to variation in ER stress induced transcriptional variation, we measured the regulatory divergence under control and TM conditions and compared the results.

**Figure 3:**
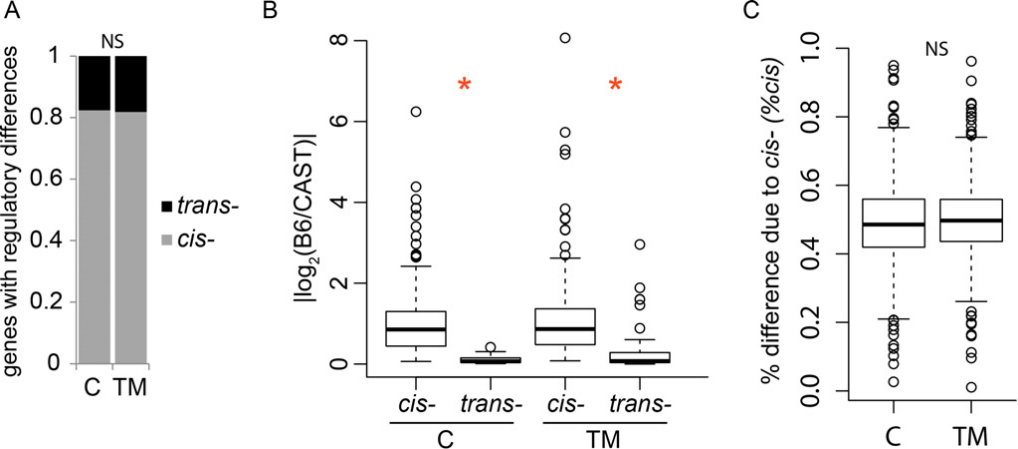
ER stress does not alter the regulatory landscape between B6 and CAST. (A) Distribution of genes that display a *cis*- or *trans*-regulatory difference between B6 and CAST under control and TM conditions. There is no significant change in the proportion of *cis*-regulatory differences between conditions. (B) The magnitude of the regulatory difference between B6 and CAST. Under both conditions, the *cis*-regulatory differences are larger than *trans*-regulatory differences (*, control: *p* < 3.746×10^-8^; TM: *p* < 0.03). (C) The amount of divergence between B6 and CAST attributable to *cis*-differences is not affected by TM. NS: not significant. C: control. TM: tunicamycin. For boxplots in (B) and (C) the boxes represent the interquartile range, the whiskers represent 1.5X interquartile range, and open circles are outliers.

Overall, we found that TM has little effect on the proportion and magnitude of *cis*-regulatory differences among strains. Under both control and TM conditions, we find a similar number of regulatory differences (*cis*- and *trans*-) between B6 and CAST—244 genes under control conditions and 264 genes under TM conditions. In fact, under both conditions, 82% of genes with regulatory differences between B6 and CAST can be attributed to a *cis*-regulatory difference (control: 201/244 genes; TM: 216/264 genes; S7 Table; Fig. 3A). The magnitude of the median *cis*-regulatory differences, under control conditions, was significantly larger than the magnitude of the median *trans*-regulatory differences (control median magnitude: *cis*: 0.52, *trans*: 0.11; *p* < 3.746×10^-8^) and this pattern was unchanged by the application of TM (TM median magnitude—*cis*: 0.50, *trans*: 0.20; *p* < 0.03) (Fig. 3B). The median amount of divergence among strains explained by *cis*-regulatory differences (% *cis*) is also unaffected by TM conditions (C: 0.49, TM: 0.50; Fig. 3C). It appears that ER stress does not alter the overall proportion of *cis*-/*trans*- regulation between B6 and CAST. A portion of the regulatory variation is also attributable to a combination of *cis*- and *trans*- variation, but these are not further considered in this study (S6 Table and S7). We found similar results for the other four F1 crosses (S2 Fig.; S6 Table, S8–S11).

This is the first study in genetically-diverse inbred mouse strains to examine the regulatory variation under ER stress. We find that most of the regulatory variation under control conditions is attributable to *cis*-regulatory variation parallels results from previous studies of regulatory variation in the CC [14] and other crosses [30]. Because of the hierarchical nature of the transcriptional response to ER stress, one might predict that ER stress regulatory variation would arise from a small number of *trans*-acting polymorphisms in the major transcription factors. Instead, just as we observed under control conditions, we find that *cis*-regulatory variation dominates under TM conditions. The fact that so few regulatory differences under TM conditions appear to arise from *trans*-acting polymorphisms, indicates polymorphisms in major transcription factors do not influence ER stress-induced transcriptional differences. This is supported by our observation that canonical ER stress transcription factors do not differ in their expression among strains (see above). Instead, genetic polymorphisms in promoters, enhancers, or other elements in individual target genes drive ER stress-induced transcriptional variation.

### ER stress uncovers unique regulatory variation among strains

**Figure 4:**
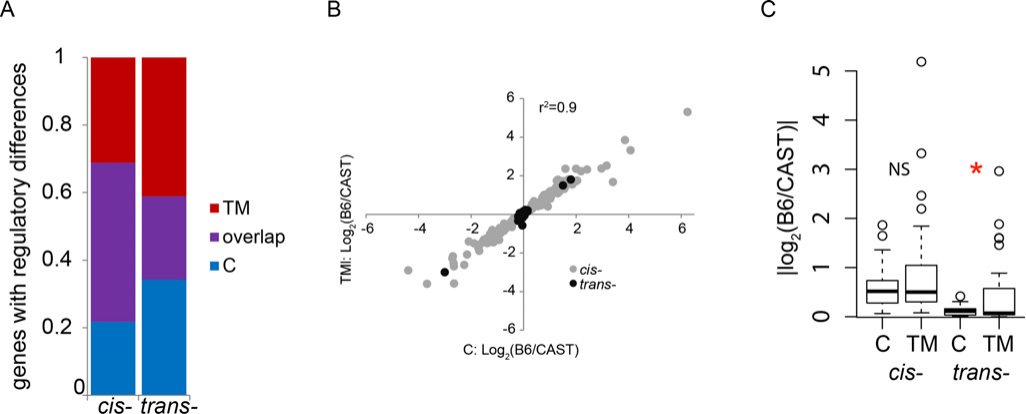
ER stress alters the genes that display regulatory differences between B6 and CAST. (A) Distribution of genes showing *cis*- or *trans*-regulatory differences found only under TM conditions, found only under control conditions, or found in both conditions. (B) Genes that show the same *cis*- or *trans*-regulatory difference under both conditions (‘purple’ genes in A) are strongly correlated in their magnitude. ER stress does not affect regulation of these genes. (C) Magnitude of the *cis*- or *trans*-regulatory differences unique to control or TM conditions. Magnitude of *cis*-regulatory differences unique to either condition is unaffected by ER stress. *trans*-regulatory differences unique to TM conditions show a small but significantly larger magnitude than *trans*-regulatory differences unique to control conditions (*, *p* < 0.05). NS: not significant; C: control; TM: tunicamycin.

We next examined whether there was overlap in the genes that showed regulatory differences between B6 and CAST, under both control and TM conditions. We find that there are a number of genes that show the same regulatory difference between B6 and CAST under both control and TM conditions. Of all the genes that show a *cis*-regulatory difference between B6 and CAST (control: 201 and TM: 216, see above), 136 genes show a *cis*-regulatory difference under both conditions (control: 67% or 136/201; TM: 63% or 136/216; S7 Table; Fig. 4A). Of the genes that show a *trans*-regulatory difference between B6 and CAST (control: 43 and TM: 48, see above), 18 genes show a *trans*-regulatory difference under both conditions (control: 42% or 18/43; TM: 38% or 18/48; S7 Table; Fig. 4A). The magnitudes of the *cis*- or *trans*-regulatory differences found under both conditions are highly correlated between the two conditions (*r^2^* = 0.9; Fig. 4B). Together this indicates that this set of *cis*- and *trans*-regulatory differences that are found under both conditions represent a group of genes whose regulatory differences between B6 and CAST are not influenced by ER stress.

It is likely that many genes harbor genetic variation affecting ER stress response, but the functional consequences of this variation are undetectable during healthy, non-stressed states. Strain differences in ER stress response might arise from the genes that show ER stress-specific regulatory variation. In particular, genes that show regulatory variation under TM conditions, and not control conditions, might reveal which ER stress pathways are most critical to strain differences in this response. Of the genes that show regulatory variation, 23% (65/281 genes) with a *cis*-regulatory difference and 34% (25/73 genes) with a *trans*-regulatory difference are unique to the control condition (S7 Table; Fig. 4A). These regulatory differences were only detected under control conditions and were not present under ER stress conditions. 28% (80/281 genes) with *cis*-regulatory differences and 41% (30/73 genes) with *trans*-regulatory differences are unique to TM conditions (S7 Table; Fig. 4A). These regulatory differences are only detectable when ER stress is induced, and absent under control conditions. Genes that show a *cis*-regulatory difference unique to either control or TM conditions do not differ in their median magnitude of expression (C: 0.52, TM: 0.50; Fig. 4C). However, there is a small but significant decrease in the median magnitude of the genes with *trans*-regulatory differences under TM compared to control conditions (C: 0.11, TM: 0.07; *p* = 0.021; Fig. 4C). The majority of genes that show regulatory differences between the two strains depend on the presence or absence of ER stress.

Genes that show evidence of differential regulation among strains may point to pathways that contribute to strain differences in ER stress response. In some cases, genes with known roles in ER stress show differential regulation among strains, and these might be useful in identifying how strains differ in canonical ER stress signaling. For example, *Gadd45a* shows a strong *cis*-regulatory difference between B6 and CAST only under ER stress conditions and not under control conditions—the B6 allele expresses 2.4 fold higher than the CAST allele. This regulatory difference is only detectable under TM conditions because *Gadd45a* shows very low expression in control conditions, but is strongly induced by ER stress. There are seven SNPs within the *Gadd45a* gene that differ between B6 and CAST, one or a combination of these polymorphisms may be responsible for this *cis*-regulatory difference. Gadd45a is induced in an ATF4-dependent manner and modulates apoptosis signaling [36]. Under stress signaling, PERK phosphorylates eIF2α which in turn results in the selective translation of ATF4 and induction of *Gadd45a* transcription [36]. Understanding how *Gadd45a* is differentially regulated will lend insight into how different genetic backgrounds might utilize *Gadd45a* function and the broader ATF4 signaling pathways to influence ER stress response. On the other hand, genes that show differential regulation and have no known function in ER stress might reveal novel ways in which different mouse strains might respond to ER stress. One such gene, *Cactin* also shows a *cis*-regulatory difference between B6 and CAST only under ER stress. The B6 *Cactin* allele expresses ~2.2 fold higher than CAST under ER stress, while under control conditions, the alleles are equally expressed. Cactin is associated with the spliceosome in Arabidopsis [37], but it is unknown if Cactin forms part of the mammalian spliceosome. Understanding how *Cactin* is differentially regulated among strains could lend insight into strain differences in splicing under ER stress conditions. In general, splicing in response to ER stress is poorly understood and provides another layer of complexity among mouse strains. Genes that show ER stress specific regulatory differences among strains may identify pathways that can be nominated as potential targets of variation in human studies.

### Shared regulatory differences

The strains utilized in the CC are genetically diverse and were chosen to maximize genetic differences among strains. Because the F1 crosses that we examined all have B6 as a common parental strain, we have the opportunity to compare how each of the five strains differs in their transcriptional regulation from B6. This analysis might reveal how the genetic architecture underlying ER stress response variation differs among strains. We hypothesized that if two (or more) F1 crosses (which share B6 as the maternal parental strain) showed evidence of the same regulatory difference in a particular gene, then the two (or more) non-B6 parental strains might differ from B6 in the same way, especially if the magnitude of the regulatory difference is equal. If the magnitude of the regulatory variation is different (a strain effect), it might indicate that different polymorphisms are driving the *cis*-regulatory differences among the F1 crosses.

**Figure 5:**
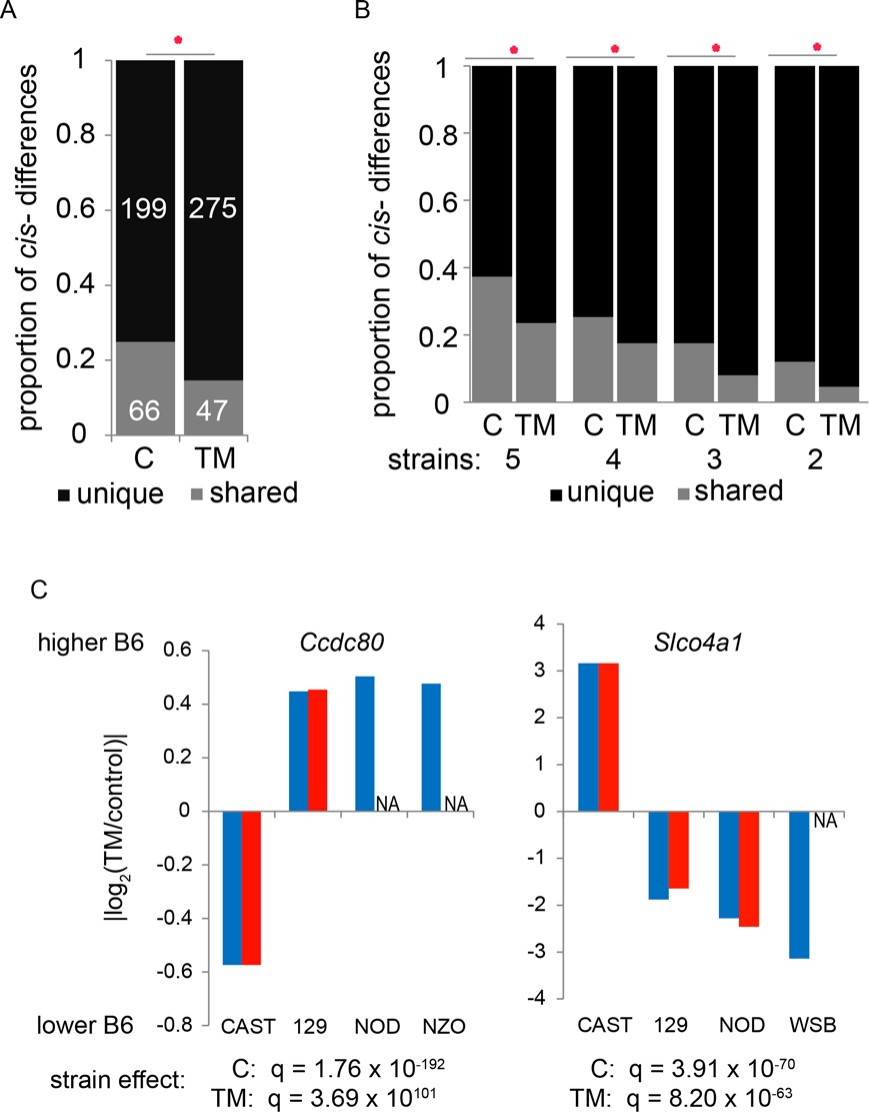
ER stress reduces the amount of shared *cis*-regulatory differences. (A) The proportion of *cis*-regulatory differences that are unique to a particular F1 combination or shared among F1 combinations. The proportion of shared *cis*-regulatory differences is significantly reduced by ER stress (*, χ^2^: *p* < 4.27×10^-9^). (B) Broken down by genes that are informative in different numbers of strains, the proportion of genes that show *cis*-regulatory differences that are unique or shared is still significantly reduced by ER stress (*, χ^2^: *p* < 0.05). (C) Examples of genes that have shared *cis*-regulatory differences. These two examples also display a significant strain effect in the magnitude of the *cis*-regulatory difference under control and TM conditions. Under ER stress, *Ccdc80* and *Slco4a1* both show reduction in the number of strains that share a *cis*-regulatory difference. NA: no detectable *cis*-regulatory difference.

We compared the genes with *cis*- or *trans*-regulatory variation in each of the five F1 crosses. Because this analysis can only be performed on informative genes of each F1 cross, we only considered genes with regulatory differences that are informative in at least two F1 crosses. Among the five F1 crosses, we find 265 genes and 322 genes with *cis*-regulatory differences under control and TM conditions, respectively. Under both conditions, the minority of *cis*-regulatory differences are shared among F1 crosses (S12 Table). Under control conditions, 25% (66/265) of genes with *cis*-regulatory differences are shared among two or more F1 combinations. Under TM conditions, 14.5% (47/322) of genes with *cis*-regulatory differences are shared among F1 combinations (Fig. 5A). TM treatment decreases the proportion of cis-regulatory differences shared among F1 combinations (From 25% to 14.5%; (χ^2^: *p* < 4×10^-7^; Fig. 5A). Even when *cis*-regulatory sharing is subdivided by number of informative strains (2, 3, 4, or 5 informative strains), TM treatment still significantly reduces the number of *cis*-regulatory differences shared among F1 combinations (χ^2^: *p* < 0.05; Fig. 5B). We find a similar pattern for *trans*-regulatory differences, where TM treatment reduces sharing between F1 combinations (S3 Fig.; S13 Table). The reduction by ER stress in the amount of shared regulatory differences among strains suggests that each strain differs from B6 in unique ways in its ER stress-induced transcriptional regulation.

The data above indicate that some genes show a signature of shared *cis*-regulatory variation among strains. However, the magnitude of most of this shared *cis*-regulatory variation differs among the F1 crosses (a strain effect). Forty genes (40/66; 61%; χ^2^, FDR 5%) under control conditions and 32 genes (32/47; 68%, χ^2^, FDR 5%) under ER stress conditions with shared *cis*-regulatory differences show a strain effect on the magnitude of the regulatory difference (S12 Table; examples in Fig. 5C). TM treatment significantly increases the proportion of shared *cis*-regulatory differences that show a strain effect (from 61% to 68%; χ^2^; *p* = 0.01). *trans*-regulatory differences also show more strain effects under TM treatment (S13 Table). Because most shared-regulatory variation differs in magnitude and that this strain effect increases with ER stress further supports the idea that induction of ER stress uncovers more genetic variation among strains.

Understanding the role of the genes that show strain differences in their *cis*-regulation may help to identity how strains respond to ER stress. *Ccdc80* is one example that shows both an ER stress-induced reduction in *cis*-regulatory sharing and a strain effect on the magnitude of those shared *cis*-regulatory differences. As mentioned above, under control conditions, *Ccdc80* shows *cis*-regulatory differences in four F1 crosses: xCAST, x129, xNOD, and xNZO. The magnitudes of these four *cis*-regulatory differences are significantly heterogeneous among strains (χ^2^, *q* < 10^-12^; Fig. 5C). The B6 allele in the xCAST cross is expressed at lower levels than the CAST allele, but the B6 allele is the more highly expressed allele in the other three F1 crosses. Under TM conditions *Ccdc80* only shows *cis*-regulatory differences in the xCAST and x129 F1 combinations and again, there is a strong effect of strain on the shared *cis*-regulatory differences (χ^2^, *q* < 10^-12^; Fig. 5C). In the case of *Ccdc80*, ER stress eliminates the *cis*-regulatory variation between B6 and NOD and between B6 and NZO. *Ccdc80* encodes a coiled-coiled domain protein which is localized to the Golgi and has been shown to relocate to the ER under certain conditions [38]. There are >100 SNPs that differ between B6 and the five strains within the *Ccdc80* gene any of these or a combination could contribute to these differences. Understanding how this type of genetic diversity contributes to ER stress response will begin to elucidate the ER stress network in each strain.

### ER stress-induced allele-specific expression

Because TM has a large influence on the *cis*-regulatory variation between B6 and the other strains, we evaluated the amount of allele-specific expression (ASE) that is influenced by TM. Because we are interested in the regulatory differences under TM conditions, we focused on those genes that showed a significant change in ASE upon TM treatment. That is, when ER stress is induced, there was a significant change in allelic ratio of expressed RNAs in the F1 cells between control and TM conditions. In all five F1 crosses, less than four percent of informative genes show a relative change in ASE upon TM treatment (S14 Table; Table 3). Often TM treatment resulted in increased expression of one allele and decreased expression of the other allele, without changing the overall expression of the gene. Less than 12% of genes in any of the F1 crosses show both a TM-induced ASE change and show TM upregulated transcript levels (Table 3).

**Table 3:** ER stress-induced allele-specific expression.

	**ASE change^#^**		**upregulated by TM**	
	**number**	%*	**number**	%**
xCAST	146	1.8	18	12.3
xNZO	113	3.9	12	10.6
xWSB	81	2.5	9	11.1
xNOD	36	2.3	3	8.3
x129	7	0.2	0	0.0

^#^ ASE is determined by comparing the read counts of each allele using the Fisher’s exact test

* percent of informative genes that show ASE change

** percent of genes that show ER stress-induced ASE

**Figure 6:**
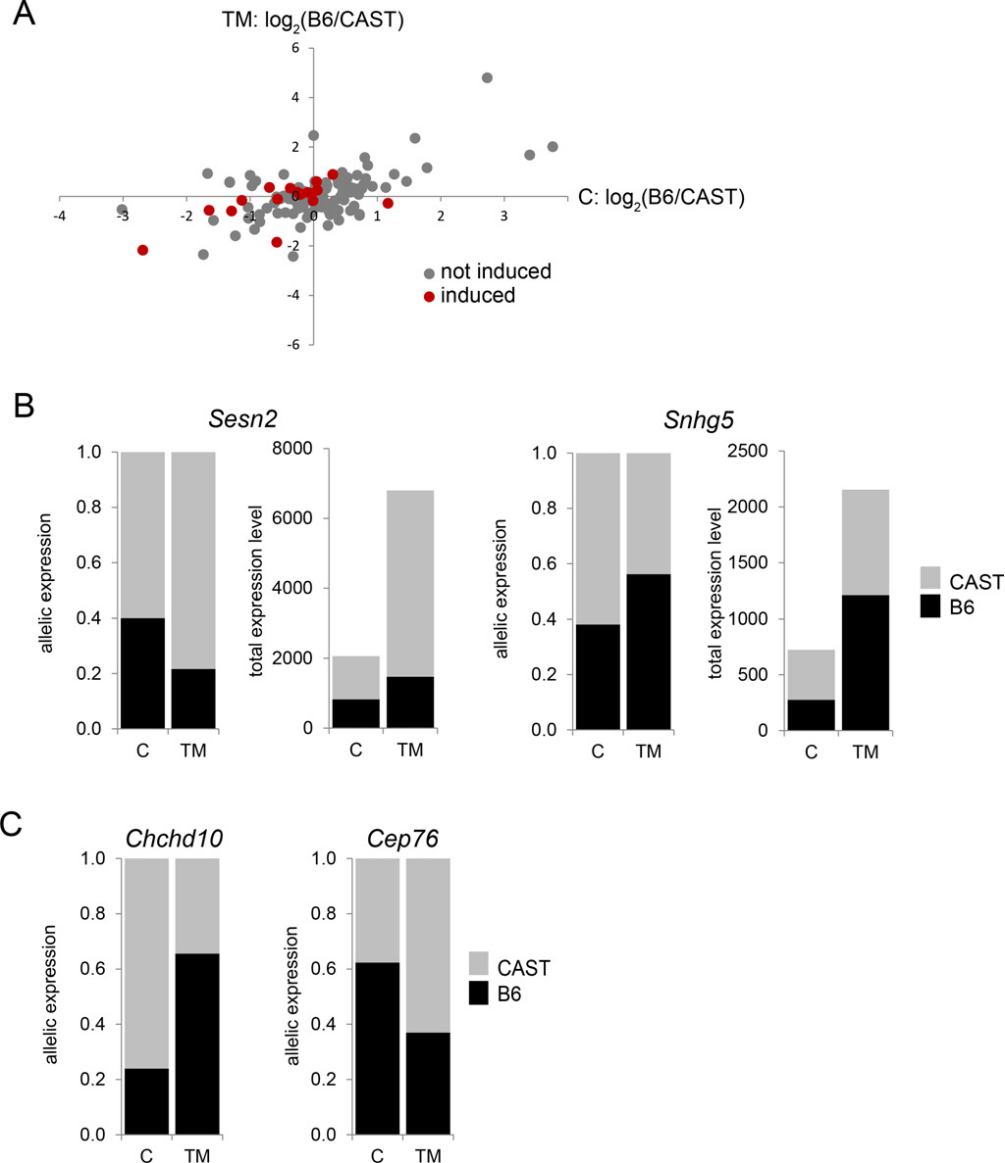
ER stress-induced allele-specific expression (ASE). (A) Genes that show ER stress-induced change in ASE, under control and TM conditions. Genes in red are genes that also show ER stress-induced increase in transcript levels. (B) *Sesn2* (Fisher’s exact; *q* < 4.30 × 10^-55^) and *Snhg5* (Fisher’s exact; *q* < 5.63 × 10^-17^) are examples of genes that show ER stress-induced change in ASE and ER stress-induced increase in transcript levels. (C) *Chchd10* (Fisher’s exact; *q* < 9.0 × 10^-4^) and *Cep76* (Fisher’s exact; *q* < 6.23 × 10^-5^) are examples of genes that show ER stress-induced change in ASE, but whose transcript is not upregulated by ER stress.

In the xCAST F1 cross, a total of 146 informative genes (1.8%) showed a change in ASE induced by TM. Sixty one genes (42%) under TM conditions show a decrease in the proportion of the B6 allele while 85 genes (58%) showed an increase in the proportion of the B6 allele (Fig. 6A). Genes that showed a change in ASE and also show increased expression under TM are of particular interest because this indicates that the two alleles in the F1 are differentially affected by TM. Of the genes that show a change in ASE, 18/146 genes (12%) also show increased transcript levels under TM conditions (Table 3). Nine of the genes have known functions in ER stress responses, including *Bip* (*Hspa5*), *Grp75* (*Hspa9*), *Trib3*, and *Sesn2*. *Sesn2*, which is involved in a variety of stress responses [39], showed the most significant change in ASE (Fisher’s exact; *q* < 4.30 × 10^-55^), where the B6 allele responds to a lower extent than the CAST allele. Under control conditions, the *Sesn2* B6 allele is expressed at 40%, but under TM conditions, B6 allelic expression is reduced to 22% (Fig. 6B). Total *Sesn2* transcript responds to TM conditions with a 3.3 fold increase. However, the B6 allele shows only a 1.7 fold increase while the CAST allele shows a 4.3 fold increase. This difference in allelic response to TM drives the change in ASE. *Snhg5* encodes a noncoding RNA and showed the second most significant change in ASE (Fisher’s exact; *q* < 5.63 × 10^-17^), where the CAST allelic response is stronger than that of B6. In this case, under control conditions, the B6 allele is expressed at 38%, but B6 allelic expression increases to 56% under TM conditions (Fig. 6B). While total *Snhg5* transcript increases by 3 fold, the B6 allele increases by 4.4 fold, but the CAST allele increases by only 2.1 fold. Again, the change in ASE is driven by a strong difference in the allelic response to TM.

The majority of genes (128 genes or 88%) that show a TM-induced ASE show no TM-induced change in total transcript level. Thirteen of the 20 genes with the most significant change in ASE do not respond to TM in total transcript abundance. For example, while total transcript level does not change, *Chchd10* shows a very large shift in ASE induced by TM (Fisher’s exact; *q* < 9.0 × 10^-4^; Fig. 6C). The *Chchd10* B6 allele is expressed at 24% under control conditions, but under TM conditions, the B6 allele is expressed at 66%. Similarly, *Cep76* shows a B6 allelic shift from 62% to 37% induced by TM (Fisher’s exact; *q* < 6.23 × 10^-5^; Fig. 6C). A change in ASE without a change in total transcript number could represent a difference in auto-regulation between the two alleles.

### Conclusions

Understanding how genetic variation impacts ER stress transcriptional response will result in a richer understanding of this basic cellular response and how its variation might influence disease severity. Because the ER stress response is under intense investigation at the molecular level, studies of its variation can be anchored within the well-studied, canonical steps and genes of this essential cellular response. Identifying how the ER stress response pathway is modulated by genetic variation serves two important purposes: 1) Identifying which canonical ER stress genes can or cannot vary in their response to ER stress and 2) Identifying novel ER stress genes that may be missed by studying only a single genetic background. By studying ER stress response in light of genetic variation, we learn how the response is buffered in different individuals and genetic backgrounds, eventually leading to potential personalized therapies that can be targeted to the specific response profile of an individual. Genetic resources in model organisms allow for the dissection of the genetic architecture behind the variable response to ER stress. Here, we took advantage of emerging resources from the mouse Collaborative Cross to study the genetic architecture of the ER stress response. This important new resource allowed us to identify variable elements of the ER stress response pathway and to uncover the genetic architecture underlying such variation.

We used TM to induce ER stress in the MEFs. While TM is commonly used to experimentally induce ER stress *in vivo* and *in vitro*, other drugs like thapsigargin and dithiothreitol are also used. These drugs have very different mechanisms of action, all resulting in ER stress as an endpoint. It is likely that using these other drugs would produce slightly different results. The results we report are specific to the concentration of TM and exposure time that we describe. These experimental conditions are in common use and the effects of TM are recognized as an acceptable experimental model of physiological ER stress. Future experiments might be warranted where the concentration and timing of exposure is altered. While these caveats limit our study to some extent, we believe, given the experimental design, the principles can be broadly applied to ER stress in general.

Our results reveal that the genetic variation underlying ER stress transcript-level responses in the mouse does not involve the major known regulators of the ER stress response. We found that on the whole, most canonical ER stress genes are not variable in TM-induced expression levels among mouse strains. Instead the strains differ most in their inflammatory response to ER stress. The finding that major regulators at the top of the signaling pathway are not variable indicates that variation arises from downstream signaling elements. If major ER stress transcription factors like Xbp1, Atf4, Atf6, or CHOP, harbored polymorphisms that affected their function, then we would expect extensive ER stress-induced *trans*-regulatory differences among strains. However, we observed the exact opposite. We found that *cis*-regulatory variation dominated the ER stress regulatory landscape, indicating that response genes themselves harbor polymorphisms that affect their response. The fact that major elements of this pathway are not variable across these “healthy” mouse strains makes sense. Previous studies show that even mice that carry heterozygous null mutations in various major ER stress genes display disease phenotypes [40]. The major ER stress response genes may not tolerate damaging polymorphisms that affect function and thus would not be a target for variation.

It is becoming increasingly clear that the effect of a particular regulatory polymorphism is context dependent [41, 42, 43]. These contexts can include, but are not limited to, environmental insults and tissue and cell types. There is likely ‘hidden’ variation that is only revealed under certain contexts. Our study demonstrates the importance of studying the role of genetic variation in different cellular contexts, like ER stress. In this study we show that induction of ER stress by TM uncovers an entire layer of *cis*- and *trans*-regulatory variation among mouse strains that is not apparent under healthy, steady state conditions. ER stress also eliminates the effect of certain regulatory variation found under healthy conditions. We also found that, for a subset of genes, the two alleles of a particular gene can display differential ER stress-induced transcriptional regulation. Regulatory variation may impact gene expression in many context-specific ways. A particular variant may affect a transcription factor binding site or might alter chromatin accessibility in a way that only affects the gene expression changes induced by ER stress. Several well defined transcription factor binding sites bind canonical ER stress transcription factors under stress conditions [18, 32, 33, 34, 35]. Because most of the regulatory variation we identified is *cis*-, these ER stress transcription factor binding sites might be where genetic variation impacts ER stress-induced transcription. SNPs or larger variants, like Copy Number Variants (CNVs), might be driving these differences we observe between strains. These polymorphisms might affect expression through various mechanisms, including epigenetic changes. We only utilized the founder strains and their F1 progeny and thus, we cannot identify the specific polymorphisms underlying these regulatory differences in this study. However, the CC recombinant inbred strains and other resources like the Diversity Outbred mice [44] are well suited for future studies aimed at mapping, identifying, and functionally studying the polymorphisms in these or other regulatory sites that might alter ER stress responses. It should also be noted that the focus of this study was on ER stress-induced gene expression variation and does not address possible differences in protein levels. Future studies aimed at identifying specific causative polymorphisms should also integrate protein abundance to understand the role of regulatory transcriptional variation on the proteome.

ER stress has been implicated in numerous diseases, including both Mendelian diseases and complex, polygenic disorders [2]. Studying genetic variation in ER stress transcriptional response in the mouse provides the opportunity to identify links between ER stress and disease that can be easily tested in the future. To identify ER stress genes that may contribute to disease, we searched for human orthologs of genes involved in Mendelian disease (OMIM) and genes involved in complex diseases (GWAS catalog). We found that 42% (89/214) of the common ER stress-responsive genes (upregulated in all eight strains) have been implicated in either Mendelian or complex diseases. Some examples include neurological diseases (gene: *Sil1*, disease: Marinesco-Sjogren Syndrome; *Zfp238*, mental retardation), diabetes (*Zfp57*, neonatal diabetes mellitus), and cancers (*Ddit3/CHOP*, myxoid liposarcoma). 39% (125/317) of ER stress-induced genes that show differences among strains have been implicated in disease. Given the enrichment of inflammation-related functions, it is not surprising that many of the diseases associated with genes that show strain differences involve inflammation (i.e. *Arhgef3*, rheumatoid arthritis; *Ifnar1*, Crohn’s disease). Other examples of diseases associated with genes with strain differences include anemia (*Slc25a38*, pyridoxine-refractory sideroblastic anemia) and neurological diseases (*Pdgfb*, basal ganglia calcification; *Bean1*, spinocerebellar ataxia 31). Understanding how these disease genes function within the context of genetic variability and ER stress will likely reveal new insight into the stress response and reveal possible roles for ER stress genes in disease pathogenesis.

This study highlights the importance of using genetic variation to study basic cellular traits like ER stress. The resources from the CC allowed us to identify novel and variably responsive ER stress genes and to uncover the complex genetic architecture that drives inter-individual transcriptional variation under ER stress. Because the ER stress pathway is well conserved, this study complements previous studies of variation in humans [7, 8] and *Drosophila* [9]. For example, the ER stress-induced expression of the mouse gene *Dhrs11* in this study and the orthologous *Drosophila* gene, *CG10962*, in a previous study [9], are both strongly influenced by genetic background. Furthermore, in *Drosophila*, a SNP in *CG10962* is strongly associated with survival under ER stress conditions [9]. This study lays the groundwork for future studies that will be aimed at identifying the genetic polymorphisms underlying the observed variation in ER stress transcriptional response and the functional consequences of such variation. The CC RI lines are perfectly suited for this type of study [14, 45, 46, 47] and will likely prove to be an excellent resource for understanding the genetic basis for variation in basic cellular traits. Understanding how genetic variation affects the ER stress transcriptional response will increase the understanding of this conserved pathway and may provide mechanistic links to disease and disease susceptibility.

## Materials and Methods

### Mouse embryonic fibroblasts

Mouse embryonic fibroblasts (MEFs) were generated from founder and F1 strains used in the Collaborative Cross (MEFs were kindly provided by Dr. David Threadgill, Texas A&M University). To eliminate individual, stochastic variation, all MEFs were generated from multiple female individuals from the same litter. Thus, each is a mix of genetically identical cells derived from multiple individual mice. F1 MEFs were derived from F1 crosses where the B6 is the maternal parent. Founder and F1 strain identities are abbreviated throughout the text (see Table 4).

**Table 4:** Abbreviations for founder and F1 strain identities.

**founder**	**abbreviation**	**F1 cross ♀ x ♂**	**abbreviation**
A/J	A	B6 X CAST	xCAST
C57BL/6J	B6	B6 × 129	x129
129S1Sv/ImJ	129	B6 X NOD	xNOD
NOD/ShiLtJ	NOD	B6 X NZO	xNZO
NZO/H1LtJ	NZO	B6 X WSB	xWSB
CAST/EiJ	CAST		
PWK/PhJ	PWK		
WSB/EiJ	WSB		

For technical reasons, we excluded the C57BL/6J X A/J and C57BL/6J X PWK F1 crosses. Excluding these two crosses does not alter the balance of this study. All MEFs were thawed at passage three or four and cells were split 1:3 every 3 days (fewer than three passages) before treatment. MEFs were maintained in DMEM containing 4.5 g/L glucose (Invitrogen), 10% fetal bovine serum, L-glutamine, and pen/strep (Invitrogen).

### Tunicamycin treatment and RNA isolation

Tunicamycin (TM) treatment of MEFs was performed using a common approach [48, 49, 50, 51, 52]. These conditions are typical of studies of ER stress in MEFs and are sufficient to elicit a strong ER stress response, without inducing apoptosis. Briefly, MEFs were plated at a concentration of 2 × 10^5^ cells/well in six-well plates. Cells were plated at least 18 h before treatment. TM stocks were prepared at 2 mg/ml in DMSO. To induce ER stress, MEFs were treated with a final concentration of 2 ug/ml of TM. Control cells were treated with 0.1% DMSO. MEFs were treated for 4 h. All genotypes and conditions were treated in parallel and in triplicate. At the end of the treatment, medium was aspirated out and Trizol (Invitrogen) was added for immediate isolation of total RNA by standard procedures.

### Illumina mRNA sequencing and mRNA-seq alignment

mRNA sequencing was performed on total RNA from 78 MEF samples (13 genotypes X 2 treatments X 3 replicates). Single-end 100 bp mRNA-seq libraries were made with 1.5 ug of total RNA using the Illumina TruSeq RNA Sample Preparation kit (Illumina Inc., CA), per manufacturer’s guidelines. The 78 samples were multiplexed and sequenced on a total of eight lanes using the Illumina HiSeq 2500 instrument. Image analysis and base calling were performed with the provided Illumina software. RNA-seq reads were aligned to the strain-specific reference mouse genome assembly using TopHat v1.4.1 [53] with three mismatches allowed. Total expression level for each transcript, measured in FPKM (Fragments Per Kilobase-pair of exon Model), was calculated based on all mapped reads [54].

### Quantification of total expression and change in expression

For both founder and F1 strains, read counts were normalized across all samples using the default normalization method (TMM) in the edgeR package in R [55, 56, 57]. For each strain and condition, principal components analysis was used to identify outlying samples. Within a strain, we required that control samples be clustered together and TM treated samples be clustered together. If a sample was not clustered with the appropriate condition, it was removed from analysis. After removal of outlying samples, there remained at least two replicates for each strain and condition combination. Remaining samples were re-normalized. TM-induced gene expression changes were assessed by comparing control vs TM treated samples using linear models with the edgeR package in R [55].

### Strain effect on expression in founder strains

For each strain, a single median expression value was calculated for each gene in the control samples. For each TM replicate, TM-induced change in expression was calculated as the log_2_ fold change of the TM expression value and the median control expression value (log_2_(TM/control)), resulting in two or three values per strain per gene. To test for a strain effect in TM-induced expression, analysis of variance (ANOVA) was used to apply a simple linear model to fold change and strain, similar to the analysis previously described [58, 59]. The TM-induce expression change (*y_i_*) for the *i* ^th^ observation of the *i* ^th^ strain was:

$$y_{ij}=\mu +strain_{i}+\varepsilon_{ij}$$

Benjamini-Hochberg correction was applied to P values to identify the set of tests with a 1% False Discovery Rate (FDR). Cluster analysis was performed with gplots in R (V.2.11.0; http://CRAN.R-project.org/package=gplots).

### Allele calls in F1 and quantification of ASE

Quality assessment of the Illumina RNA-seq reads was performed using FastQC software (http://www.bioinformatics.babraham.ac.uk/projects/fastqc/) for all samples in the five F1 crosses. Positions with low Q-score at the end of the reads were trimmed by FASTX-Toolkit (http://hannonlab.cshl.edu/fastx_toolkit/index.html). Adapter sequences were trimmed by Trimmomatic software [60]. As recommended by Munger et al., 2014 [47], the cleaned reads were mapped to both paternal (CAST, 129S1, NOD, NZO and WSB) and maternal genomes (B6) using TopHat v1.4.1 [53, 54] allowing three mismatches. Local realignment over indel positions was performed with GATK software [61], and only uniquely mapped reads were included in the final BAM files that were submitted for SNP calling and allele count summary. Based on the SNP and genome information from the Sanger Mouse Genomes Project (http://www.sanger.ac.uk/resources/mouse/genomes/), parental allele counts were summarized using SAMtools [53, 54] at SNP positions with coverage of four or more. Problematic SNP positions, such as those that did not display 100% monoalleic expression in parental crosses, exhibited a third allele, were in repetitive regions, were near an indel position, or occurred at exon-intron junctions, were filtered out using custom scripts [62] resulting in 12,4587, 32,632, 27,686, 35,397 and 33,775 high-quality covered SNPs for xCAST, x129S1, xNOD, xNZO and xWSB crosses respectively. From the autosomal, X-linked and mitochondrial SNPs, all the F1 RNA-seq data matched the expected genotype identities.

The SNPs were annotated using SnpEff [63] on Ensembl v64 (www.ensembl.org). Only exonic SNPs based on Ensembl gene models were used to generate per-transcript allele counts summarized over multiple SNPs in the same transcript. The averaged counts from paternal and maternal genome alignments were used. To quantify allele-specific expression, we calculated the ratio of the number of reference allele-containing reads divided by the total coverage within a transcript for autosomal genes covered with informative SNPs in each cross [64]. To reduce the inflated inter-replicate variability of allelic expression ratio [62] and increase the SNP coverage, we combined all replicates and restricted our analysis only to genes with exonic SNP coverage >20 for *cis*-/*trans*- analysis and >50 for ASE analysis. We were able to estimate allele-specific expression ratios for ~9100 genes in xCAST cross, ~4700 genes in xWSB cross, and ~3500 genes in each of the other three F1 crosses. To detect significant changes of allelic expression between control and TM treated samples, we applied Fisher’s Exact Test on the reference and alternative allele counts in control and treated samples, followed by FDR correction. The slight over-dispersion of the data could inflate the false positive rate of the Fisher Exact test, but the rank-order of significance is preserved and because we are comparing the same genes in control and treated samples we believe that the reported false discovery rates are reasonable.

### Inference of *cis*- and *trans*-regulation

For purposes of biological inference, we binned the genes into categories of *cis*-, *trans*-, or a combination of *cis*- and *trans*- regulation following a method previously described in McManus et al. 2010 [29]. A more detailed quantitative analysis that partitions the variance in regulation will be applied to an expanded data set in the future. *cis*- and *trans*-regulation among strains was inferred using a hierarchical statistical analysis in R. We applied a very conservative significance threshold at every level of this analysis (FDR = 0.1%). For each strain, control and TM conditions were analyzed separately. The analysis proceeded as follows:

1) Differential expression in parental (P) and F1 data sets was analyzed using the binomial exact test followed by FDR correction.
2) For all genes where there was differential expression between P strains or between parental alleles in the F1, Fisher’s exact test was used to compare the strain-specific expression ratios in the P and F1 samples, followed by FDR correction. A significant difference in the ratios of P vs F1 samples is considered a *trans*-regulatory difference.

As mentioned above, the Fisher’s exact test assumes independent sampling of reads, but the nature of RNA-seq experiments often inflates the error, potentially leading to false positives [62]. We avoid this problem by taking every effort to generate libraries of high complexity, which we verified using the replicates to directly test overdispersion. Only genes showing *cis*- or *trans*- regulation are discussed in the text, but genes in other categories are presented in the supplement (S7 Table–S11). Based on the results of the analysis in (1) and (2), genes were partitioned into the following categories: *cis*-, *trans*-, *cis- + trans*-, *cis- x trans*-, compensatory, conserved, and ambiguous (See Table 5).

**Table 5:** Inference of *cis*- and *trans*- regulation.

	P*	F1*	***trans^^^***	**P vs F1^@^**
***cis*-**	Yes	Yes	No	NA
***trans*-**	Yes	No	Yes	NA
***cis*- + *trans*-**	Yes	Yes	Yes	same
***cis*- *x trans*-**	Yes	Yes	Yes	opposite
**compensatory**	No	Yes	Yes	NA
**conserved**	No	No	No	NA
**ambiguous**	all other genes			

* See methods: Differential expression in parental strains (P) or alleles (F1) based on analysis from (1).

^^^ See methods: Change in ratio based on analysis from (2).

^@^ See methods: log_2_ strain specific ratio is either the same or opposite in P and F1

### Identification of shared regulatory difference

Only genes that are informative between two or more F1 strains were considered. A gene was considered to have a shared regulatory difference if it displayed either a *cis*- or *trans*-regulatory difference between two or more F1 crosses. The ratio of the allelic expression (magnitude) in the F1 crosses was tested for a strain effect by chi-squared (χ^2^) analysis.

### Bioinformatics functional analysis

All Gene Ontology (GO) analysis was performed using DAVID [65, 66]. Transcription factor binding sites were identified using mouse single site analysis (version 2) in oPOSSUM [67]. Searches were limited to 2000 base pairs up- and downstream of the transcription start site. Genes involved in human disease were identified by using Online Mendelian Inheritance in Man (OMIM; www.omim.org) and the GWAS Catalog (https://www.genome.gov/26525384). Literature searches were performed in PubMed (http://www.ncbi.nlm.nih.gov/pubmed). Visualization of regulatory networks was performed in Cytoscape [68].

## Supporting Information

S1 Fig Identify of genes and values from the cluster analysis in Fig. 2C. (TIF)

S2 Fig Proportion of *cis*- and *trans*- regulatory differences in the five F1 crosses. (TIF)

S3 Fig ER stress reduces the amount of shares *trans*-regulator differences. (A) The proportion of *trans*-regulatory differences that are unique to a particular F1 combination or shared among F1 combinations. The proportion of shared *trans*-regulatory differences is significantly reduced by ER stress (*, x^2^: P<10^-4^). (B) Broken down by genes that are informative in different number of strains, the proportion of genes that show *trans*-regulatory differences that are unique or shared is still significantly reduced by ER stress (*, x^2^: P<0.05).
(TIF)

S1 Table ER stress induced genes. (XLSX)

S2 Table Strain effect of ER stress induced genes. (XLSX)

S3 Table Identity of top 100 ER stress induced genes with strain effect. (XLSX)

S4 Table Pairwise correlations of inflammation genes. (XLSX)

S5 Table Informative genes in F1s. (XLSX)

S6 Table Number of regulatory differences. (XLSX)

S7 Table B6×CAST F1. (XLSX)

S8 Table B6×129 F1. (XLSX)

S9 Table B6×NZO F1. (XLSX)

S10 Table B6×NOD F1. (XLSX)

S11 Table B6×WSB F1. (XLSX)

S12 Table Shared *cis*- effects. (XLSX)

S13 Table Shared *trans*- effects. (XLSX)

S14 Table Allele specific expression. (XLSX)
