# Supplementary figures and images for: The Genetic Architecture of the Genome-Wide Transcriptional Response to ER Stress in the Mouse
Source: PLoS Genet. 2015 Feb 4;11(2):e1004924. doi: 10.1371/journal.pgen.1004924 (PMC4412289; doi:10.1371/journal.pgen.1004924)

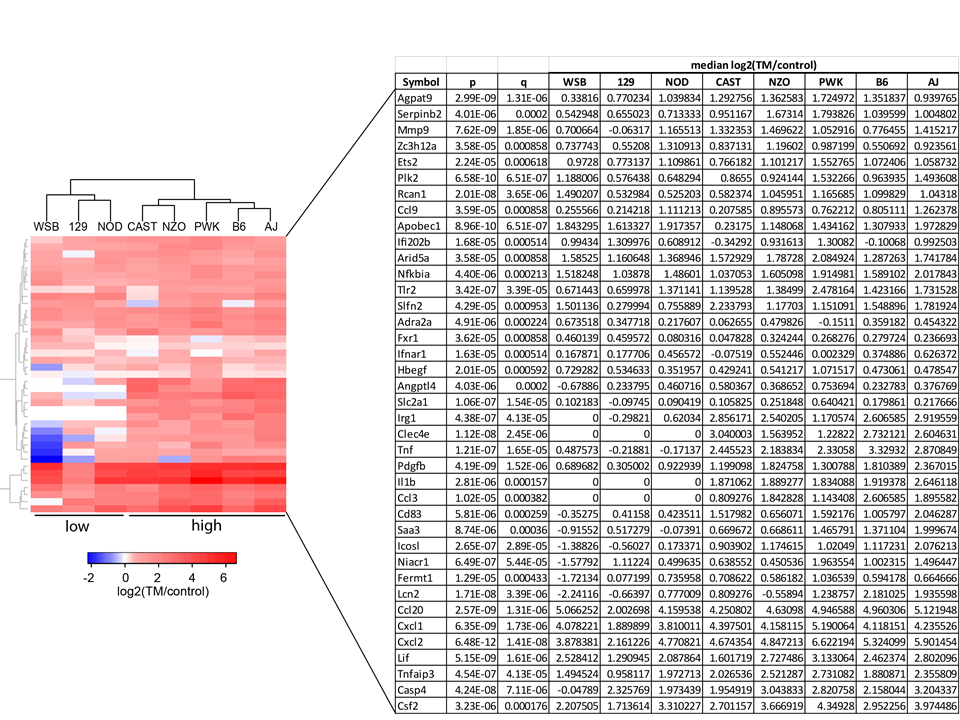

Supplement: S1 Fig — (TIF) [file pgen.1004924.s001.tif]

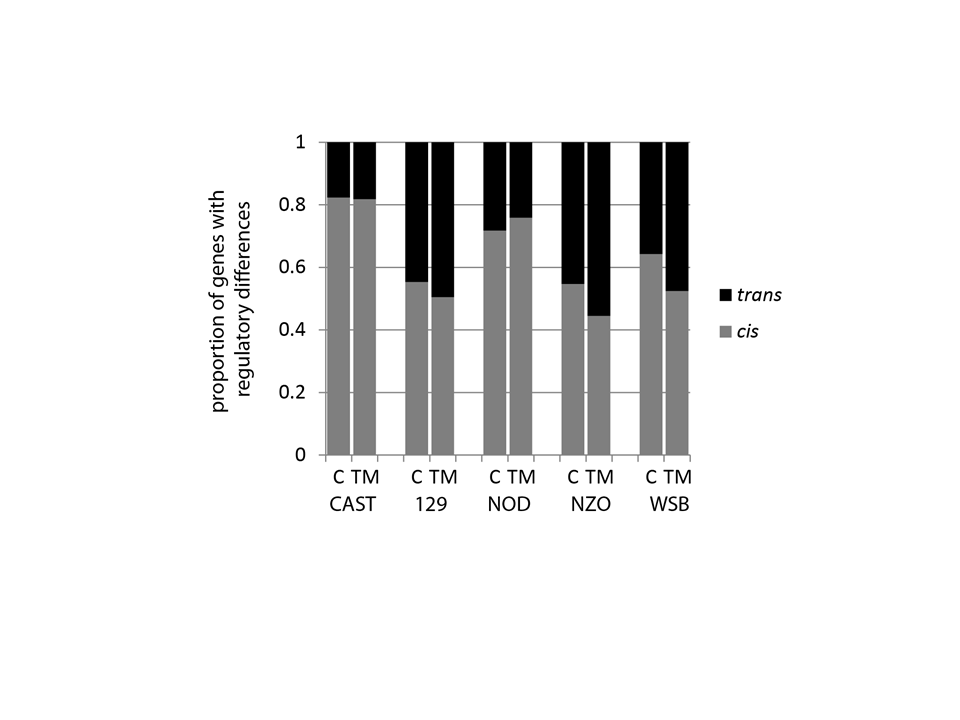

Supplement: S2 Fig — (TIF) [file pgen.1004924.s002.tif]

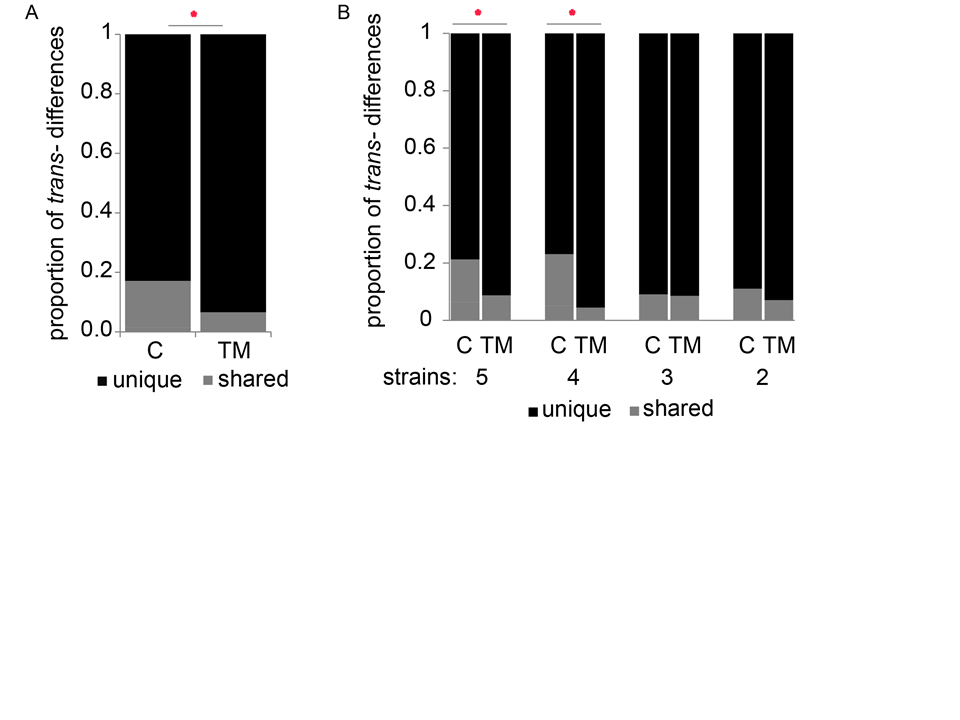

Supplement: S3 Fig — (A) The proportion of trans-regulatory differences that are unique to a particular F1 combination or shared among F1 combinations. The proportion of shared trans-regulatory differences is significantly reduced by ER stress (*, x2: P<10-4). (B) Broken down by genes that are informative in different number of strains, the proportion of genes that show trans-regulatory differences that are unique or shared is still significantly reduced by ER stress (*, x2: P<0.05). (TIF) [file pgen.1004924.s003.tif]
